# wQFM-DISCO: DISCO-enabled wQFM improves phylogenomic analyses despite the presence of paralogs

**DOI:** 10.1093/bioadv/vbae189

**Published:** 2024-11-27

**Authors:** Sheikh Azizul Hakim, Md Rownok Zahan Ratul, Md Shamsuzzoha Bayzid

**Affiliations:** Department of Computer Science and Engineering, Bangladesh University of Engineering and Technology, Dhaka 1205, Bangladesh; Department of Computer Science and Engineering, Bangladesh University of Engineering and Technology, Dhaka 1205, Bangladesh; Department of Computer Science and Engineering, Bangladesh University of Engineering and Technology, Dhaka 1205, Bangladesh

## Abstract

**Motivation:**

Gene trees often differ from the species trees that contain them due to various factors, including incomplete lineage sorting (ILS) and gene duplication and loss (GDL). Several highly accurate species tree estimation methods have been introduced to explicitly address ILS, including ASTRAL, a widely used statistically consistent method, and wQFM, a quartet amalgamation approach experimentally shown to be more accurate than ASTRAL. Two recent advancements, ASTRAL-Pro and DISCO, have emerged in phylogenomics to consider GDL. ASTRAL-Pro introduces a refined quartet similarity measure, accounting for orthology and paralogy. On the other hand, DISCO offers a general strategy to decompose multi-copy gene trees into a collection of single-copy trees, allowing the utilization of methods previously designed for species tree inference in the context of single-copy gene trees.

**Results:**

In this study, we first introduce some variants of DISCO to examine its underlying hypotheses and present analytical results on the statistical guarantees of DISCO. In particular, we introduce DISCO-R, a variant of DISCO with a refined and improved pruning strategy that provides more accurate and robust results. We then demonstrate with extensive evaluation studies on a collection of simulated and real data sets that wQFM paired with DISCO variants consistently matches or outperforms ASTRAL-Pro and other competing methods.

**Availability and implementation:**

DISCO-R and other variants are freely available at https://github.com/skhakim/DISCO-variants.

## 1 Introduction

Inferring species trees from gene trees sampled throughout the whole genome is a fundamental problem in molecular evolutionary biology. However, this task is complicated by gene tree heterogeneity (or discordance), where different genes may have distinct evolutionary histories. Gene tree heterogeneity can arise due to various biological processes, including incomplete lineage sorting (ILS), gene duplication and loss (GDL), etc. (Warnow 2017). Numerous methods have been proposed for species tree inference, including co-estimation of gene trees and the species tree (Liu 2008, Heled and Drummond 2010, Boussau *et al.* 2013, Szöllősi *et al.* 2015), and species tree inference directly from sequence data (Chifman and Kubatko 2014). However, the most scalable and popular approach to date remains a two-step process, where gene trees are first inferred independently from sequence data and then combined using summary methods that attempt to estimate species trees by summarizing the input gene trees under a model of gene tree discordance.

Many summary methods have been proposed to target ILS. Some of them are statistically consistent under the multi-species coalescent (MSC) model (Snir and Rao 2010, Zhang 2011, Bayzid and Warnow 2012, Chifman and Kubatko 2014, Mirarab *et al.* 2014, Reaz *et al.* 2014, Avni *et al.* 2015, Islam *et al.* 2020, Mahbub *et al.* 2021, Mim *et al.* 2023). Maximizing quartet consistency (MQC) is one of the leading optimization criteria for estimating statistically consistent species trees from gene trees in the presence of ILS (Snir and Rao 2010, Mirarab *et al.* 2014, Reaz *et al.* 2014, Avni *et al.* 2015, Mahbub *et al.* 2021). MQC seeks a species tree consistent with the largest number of quartets induced by the set of gene trees. ASTRAL is arguably the most commonly used quartet-based summary method (Mirarab *et al.* 2014). Given a set of unrooted gene trees, ASTRAL uses a dynamic programming approach to solve the *NP*-hard optimization problem of finding the species tree that agrees with the largest number of quartet trees induced by the set of gene trees. A different method involves deducing individual quartets and then combining them into a cohesive species tree in a divide-and-conquer fashion, either with or without weights, e.g., Quartets Max-Cut (QMC) (Snir and Rao 2010), Quartets Fiduccia–Mattheyses (QFM) (Reaz *et al.* 2014), Weighted Quartets Max-Cut (wQMC) (Avni *et al.* 2015), Weighted Quartets Fiduccia–Mattheyses (wQFM) (Mahbub *et al.* 2021), etc. While ASTRAL is an exact dynamic programming algorithm statistically consistent under the MSC model, wQMC and wQFM are heuristic-based methods, and no result on statistical consistency exists in the literature. However, through extensive simulation studies, wQFM has been shown to have consistently outperformed wQMC and ASTRAL (Mahbub *et al.* 2021, 2022), even when gene trees are incomplete (Mahbub *et al.* 2022). These methods were initially proposed to address gene tree heterogeneity due to ILS and are thus applicable to single-copy gene trees.

GDL events introduce paralogs, which are genes originating from a common ancestor through duplication events, in addition to orthologs, which arise from speciation events (Fitch 2000, Yang and Smith 2014). Consequently, gene families may have multiple genes with the same species labels, resulting in multi-copy gene trees. However, most summary methods require genes with at most one copy in each species, so they cannot be used directly in the presence of GDL. There have been a few summary methods developed to estimate species trees from multi-copy gene trees by addressing gene duplications and losses and without requiring orthology determination, namely PHYLDOG (Boussau *et al.* 2013), iGTP (Chaudhary *et al.* 2010), DupTree (Wehe *et al.* 2008), DynaDup (Bayzid *et al.* 2013, Bayzid and Warnow 2018, Bayzid 2023), MulRF (Chaudhary *et al.* 2013), FastMulRFS (Molloy and Warnow 2020), Guenomu (De Oliveira Martins *et al.* 2016), ASTRAL-Pro (Zhang *et al.* 2020), and SpeciesRax (Morel *et al.* 2022). Most of these methods (Wehe *et al.* 2008, Chaudhary *et al.* 2010, Bayzid *et al.* 2013, Chaudhary *et al.* 2013, Bayzid and Warnow 2018) are parsimony-based approaches where a species tree is estimated by minimizing a particular distance between the species tree and given set of gene trees (e.g., the total number of duplication and loss events). In particular, ASTRAL-Pro is a quartet-based species tree inference method that takes a set of multi-copy gene family trees and aims to compute a species tree that maximizes the total similarity to the input gene trees, using a new measure of quartet similarity that accounts for orthology and paralogy (Zhang *et al.* 2020).

Another approach to tackle multi-copy gene family trees resulting from GDL events is to decompose multi-copy gene trees into single-copy gene trees (Hejnol *et al.* 2009, Dunn *et al.* 2013). Wilson *et al.* introduced the DISCO algorithm to decompose multi-copy gene trees into single-copy gene trees, allowing the utilization of methods designed for single-copy gene trees in handling multi-copy gene trees (Willson *et al.* 2022). To obtain a rooted tagged (duplication/speciation) multi-labeled tree, DISCO utilizes the tagging and rooting heuristic utilized by ASTRAL-Pro and uses a pruning strategy to produce single-copy gene tress from multi-copy gene trees. ASTRAL-Pro and ASTRAL-DISCO (ASTRAL on the output set of DISCO) are statistically consistent when either ILS or GDL is active (Zhang *et al.* 2020, Willson *et al.* 2022). Many successful summary methods (ASTRAL, wQFM, MP-EST, etc.) were designed to model ILS and are therefore, suitable for only single-copy gene trees. Thus, any fundamental improvements of DISCO and its applications to enhance the capabilities of highly accurate methods such as wQFM in modeling both orthology and paralogy, thereby making them suitable for multi-copy gene trees will be of great interest for estimating species trees from multi-locus data in the presence of GDL.

This study entails the following key contributions. We first examine some underlying hypotheses regarding the pruning and tagging strategies of DISCO combined with both ASTRAL and wQFM and propose DISCO-R (DISCO with a **R**efined pruning strategy that **R**egrafts clades that original DISCO may have missed), which resulted in more accurate and robust results. For the first time, we will apply wQFM in the multi-copy gene tree setting and establish its experimental superiority over other competing methods, including ASTRAL-Pro. Our findings from an extensive evaluation study highlight the effectiveness of combining wQFM with DISCO variants and demonstrate its superior performance in species tree estimation from multi-copy gene trees. We also extend theoretical guarantees of statistical consistency of ASTRAL combined with DISCO by showing that ASTRAL-DISCO is statistically consistent under the DLCoal (Duplication, Loss, and Coalescence) model—a model to incorporate both ILS and GDL (Rasmussen and Kellis 2012).

## 2 Background

We provide the essential definitions in this section, with a more in-depth discussion of the underlying models and existing algorithms in Section S1 of the Supplementary Material.

A ‘species tree’ represents the evolutionary history of a group of species. A ‘locus tree’, obtained from a species tree by running the duplication/loss process top-down along its edges, represents a duplication-loss history of a particular gene. A locus tree node can be termed as ‘speciation’ if such a node corresponds to a speciation event/node in the species tree or as ‘duplication’ if such a node corresponds to creating a new locus. The ‘gene tree’ is obtained from a locus tree by running the bounded multi-species coalescent (b-MSC) process bottom-up along the edges of the locus tree. The duplication/loss process and the b-MSC process are jointly referred to as the DLCoal model.

Given a model of evolution, any species tree estimation method is ‘statistically consistent’ under the specified model if the method is guaranteed to produce the true species tree when sufficiently many error-free gene trees are provided as input.

## 3 Statistical consistency of ASTRAL-DISCO under the unified DLCoal model

ASTRAL-DISCO was shown to be statistically consistent when the cause of discordance is only GDL, provided that the rooting and tagging algorithm of ASTRAL-Pro is correct (Willson *et al.* 2022). ASTRAL-ONE was also proved to be statistically consistent under GDL (Legried *et al.* 2021). The ASTRAL-ONE pipeline consists of two stages. First, given a collection of multi-copy gene trees, a single-copy gene tree is obtained from each multi-copy tree by randomly sampling one gene from each species. Then, ASTRAL is run on the single-copy gene trees. Markin and Eulenstein (2021) extended the results of Legried *et al.* (2021) when ILS is also involved besides GDL. Under the unified (DLCoal) process (Rasmussen and Kellis 2012), the following theorem holds (Markin and Eulenstein 2021).

Theorem 1. (Markin and Eulenstein 2021) *Let S be a species tree with four leaves that displays quartet* AB|CD*, and let G be a gene tree that evolved in S according to the DLCoal process. If one picks genes* a,b,c,d*(that correspond to species* A,B,C,D, *respectively) uniformly at random (assuming they exist) from G, then* P(ab|cd∈G)>P(ac|bd∈G)=P(ad|bc∈G).

When the inequality in Theorem 1 is satisfied, Legried *et al.* (2021) proved that ASTRAL-ONE is a statistically consistent estimator as presented in Theorem 2.

Theorem 2. (Legried *et al.* 2021) (rephrased). *Let S be a species tree with four leaves that displays quartet* AB|CD*, and let* G*be an arbitrarily large collection of gene trees over the species in S. If one picks genes* a,b,c,d*(that correspond to species* A,B,C,D, *respectively) uniformly at random (assuming they exist) from any* G∈G*and the resultant quartet q in G is more likely to match the species tree configuration than the other two, i.e.*, P(q=ab|cd)>P(q=ac|bd)=P(q=ad|bc)*, then ASTRAL-ONE almost surely returns S when run with* G.

We extend the previous results to show the statistical consistency of ASTRAL-DISCO under the unified DLCoal model (Theorem 3).

Theorem 3. *ASTRAL-DISCO is statistically consistent under the DLCoal process, provided that the input gene trees are correctly rooted and tagged.*

Proof. By assumption, each gene tree is obtained by running the DLCoal process over the true species tree, and the rooting and tagging are correct. Hence, each duplication node in the gene tree marks the creation of a new locus. We consider that the subtree to be kept follows the parent locus (mother duplicate), and the subtree to be pruned follows the novel locus (daughter duplicate). Therefore, each resultant DISCO tree represents the history of a particular locus, which obeys the bounded-MSC model (which is a DLCoal process with duplication and loss rate being zero). If one picks genes a,b,c,d (that correspond to species A,B,C,D, respectively, assuming they exist) from any resultant DISCO tree *G*, then by Theorem 1, P(ab|cd∈G)>P(ac|bd∈G)=P(ad|bc∈G). Since a resultant DISCO tree is single-copy, there is only one quartet *q* induced by genes a,b,c,d, which can be either ab|cd, ac|bd, or ad|bc. So, P(q=ab|cd)>P(q=ac|bd)=P(q=ad|bc). Hence, by Theorem 2, ASTRAL-ONE on the resultant DISCO trees is statistically consistent under the DLCoal process. Since DISCO trees are single-copy trees, running ASTRAL on DISCO trees and ASTRAL-ONE on DISCO trees are equivalent, and the theorem follows.□

ASTRAL was designed to solve the Maximum Quartet Support Species Tree (MQSST) problem, which aims to find the species tree that agrees with the largest number of quartet trees induced by the set of gene trees (Mirarab *et al.* 2014). Since ASTRAL, in its exact and heuristic version, exactly solves the MQSST problem when there are many error-free gene trees, all the statistical consistency results for ASTRAL will hold for any other algorithm that accurately solves MQSST. wQFM was designed to solve the WMQC problem, which takes a set Q of weighted quartets on a set S of taxa as input and aims to find the tree *T* on S such that the total weight of the quartets in Q that are consistent with *T* is maximized. If the quartet amalgamation technique is proper and the weights of the input quartets are computed based on GTF, then the unique solution to the WMQC problem (the problem wQFM tries to solve heuristically) is a statistically consistent estimator of the true species tree under the MSC model (Mahbub *et al.* 2021). Following the same argument as above, we can conclude the following theorem.

Theorem 4. *If the quartet amalgamation technique is proper and the weights of the input quartets are computed based on GTF of the trees decomposed by DISCO, then the unique solution to the* WMQC*problem is a statistically consistent estimator of the true species tree under the DLCoal model.*

## 4 DISCO-R: a refined pruning strategy using regrafting

In this study, we tried to investigate some variants of DISCO and empirically showed that DISCO is quite robust. In particular, we propose an improved pruning strategy and call this variant DISCO-R. Zhang *et al.* (2020) introduced the concept of speciation-driven quartets (SQ) to formulate the ASTRAL-Pro algorithm. ASTRAL-Pro roots and tags using the maximum parsimony principle with equal penalties for duplication and loss, and no penalty for ILS. A node is termed as ‘duplication’ if some species can be reached from both children of the node and as ‘speciation’ otherwise. A quartet ab|cd is defined as a speciation-driven quartet (SQ) if all four genes are contained in different species and the least common ancestor of either a or b with either c or d is tagged as speciation. The authors claimed that only SQs contain information about speciation (Zhang *et al.* 2020). Since DISCO uses the rooting and tagging heuristic introduced by ASTRAL-Pro (Algorithm S1 in the Supplementary Material), the quartets in the resultant DISCO trees are all marked as SQs and considered by ASTRAL-Pro (Willson *et al.* 2022). However, DISCO does not consider all speciation-driven quartets. Here, we introduce a new pruning heuristic, DISCO-R, that provably includes more SQs than DISCO. We empirically show that DISCO-R performs at least as good as or better than DISCO.

**Figure 1.**
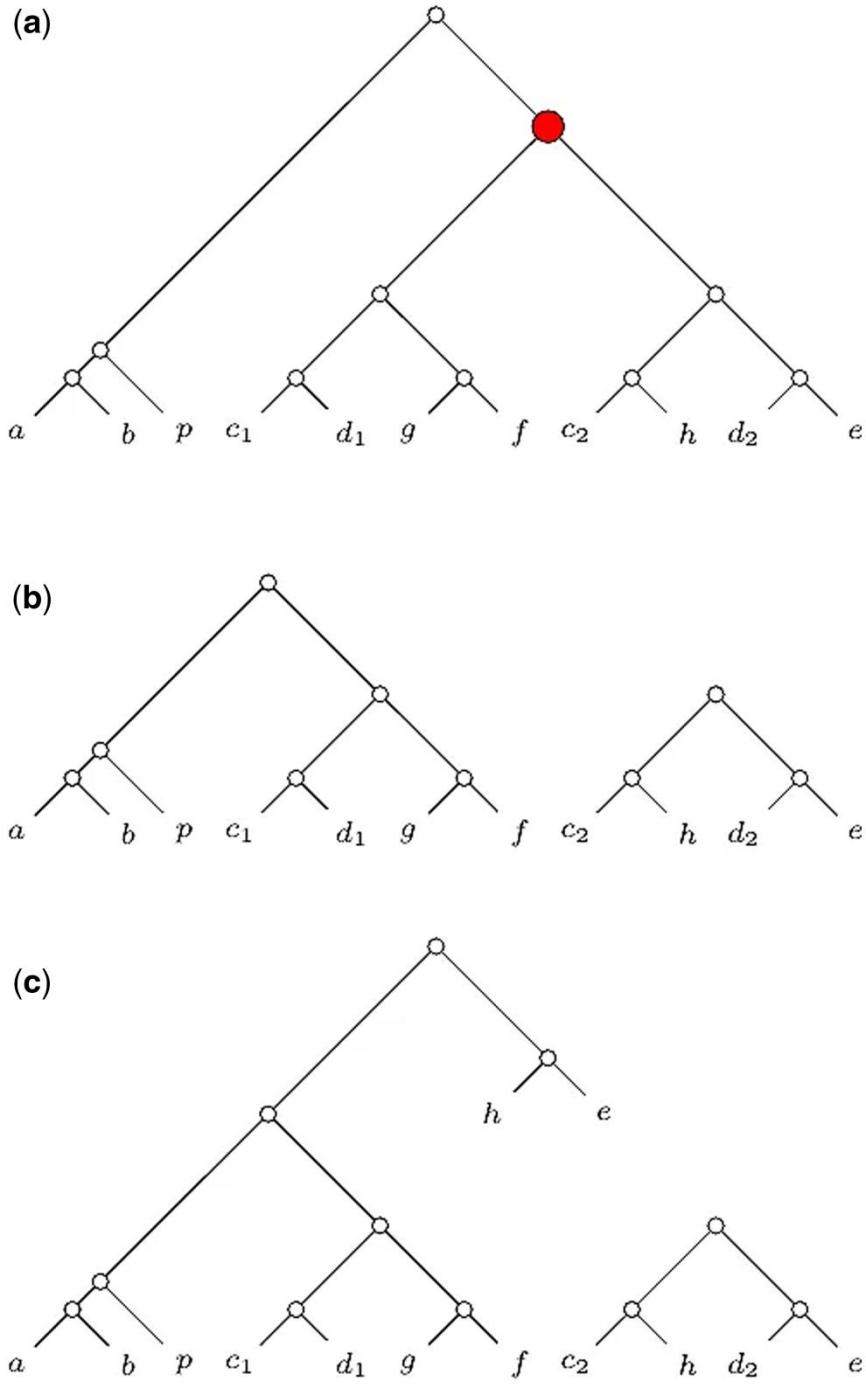
(a) A multi-copy gene tree, with a duplication node marked as red. (b) Its decomposition output with DISCO. (c) Its decomposition output with DISCO-R.

Let us consider a multi-copy gene tree *G* in Fig. 1. We differentiate the instances of the same species by a numerical subscript. There is one duplication node, which we mark as red. Since both subtrees have the same number of leaves, DISCO could prune any of the subtrees. Without loss of generality, let us assume that the subtree $\left((c_{2},h),(d_{2},e)\right)$ was pruned. When fed with *G*, DISCO produces the two trees in Fig. 1. All quartets in the output of DISCO trees are speciation-driven. However, the output misses some SQs such as $ac_{2}|d_{2}e,\;ah|d_{2}e,\;ab|pe,\;ab|ph$, etc. Meanwhile, ASTRAL-Pro takes into consideration all SQs of *G*.

We modify DISCO to include the quartets that DISCO usually misses, i.e., in our example in Fig. 1, we want to include the quartets ab|pe and ab|ph. In our new method DISCO-R, at a duplication node, we prune a subtree as usual. In addition, we take a subtree of the pruned subtree, including only those species not present in the backbone tree, and reattach it in the place of the duplication node in the backbone tree. Then we contract all degree-2 nodes. Algorithm 1 represents the workflow of both DISCO and DISCO-R.

The output of DISCO-R when run with *G* is shown in Fig. 1. This scheme successfully incorporates all SQs retained by DISCO and some other speciation-driven quartets, such as ab|pe, ab|ph, ab|he, bp|he, etc. However, some other SQs, such as $ac_{2}|d_{2}e,\;ah|d_{2}e$, etc., are still missing in DISCO-R. Moreover, DISCO-R introduces quartets like, $ae|c_{1}d_{1},\;ad_{1}|he$, etc., which are present in *G*, but are not speciation-driven quartets. In brief, the quartets generated by DISCO are SQs, but not all SQs are covered by DISCO output trees. DISCO-R might cover more SQs than DISCO, but DISCO-R can introduce some non-SQs, too. ASTRAL-Pro claims that these non-SQs do not convey speciation-related information (Zhang *et al.* 2020). However, we experimentally find that DISCO-R, which considers potentially more SQs and some non-SQs, can perform better than DISCO, which does not use any non-SQ at all (check Experiment 1). Moreover, it is possible to construct a collection of gene trees $C=\{G_{i}\}$ such that one species is entirely missing from $C\prime =\underset{i}{\cup }S_{G_{i}}$, where $S_{G_{i}}$ is the set of species whose genes are contained in *G_i_*. Such events cannot occur in DISCO-R because we do not prune any non-duplicate leaf. Such an example, adapted from (Willson *et al.* 2022), is shown in Fig. S1 of the Supplementary Material.

**Algorithm 1**. The pseudocode of DISCO/DISCO-R. Lines 8–13 are used only for DISCO-R.

**Require:** A rooted multi-copy gene tree *g* with each node tagged as duplication/speciation

**Ensure:** A set of single-copy gene trees *S*

1: S←∅

2:  **for** node *v* in the postorder traversal of *g* **do**

3:   **if** *v* is a duplication node **then**

4:    $g_{v_{l}},\;g_{v_{r}}\leftarrow$ be the left and right subtrees of *v*

5:    $g_{v}\leftarrow g_{v_{l}}$**if**$\left|\text{leaves}(g_{v_{l}})|>|\text{leaves}(g_{v_{r}})\right|$**else**$g_{v_{r}}$

6:    delete *g_v_* from *g*

7:    $S\leftarrow S\cup \{g_{v}\}$

8:    **if** DISCO-R is being used **then**

9:     L← the set of species denoted by the leaves in *g*

10:     delete all leaves *l* from *g_v_* where *l* denotes a species from L

11:     attach the root of *g_v_* in the place of *v* in *g*

12:     contract the degree-2 nodes in *g*

13:    **end if**

14:   **end if**

15:  **end for**

16: S←S∪{g}

## 5 Experimental studies

### 5.1 Datasets

#### 5.1.1 Simulated dataset

We analyzed a 25-taxa dataset simulated and analyzed in the ASTRAL-Pro study (Zhang *et al.* 2020). Here, the number of species is 25, and the number of replicates in each model condition is 50. Each replicate has 1000 true gene trees simulated under the DLCoal model. The duplication rate ($\lambda_{+}$), which is also equal to the loss rate ($\lambda_{-}$), is $4.9\times 10^{-10}$. Gene trees have an ILS level of [60%,80%] for the default model. We performed most of our experiments with varying duplication rates from 0 to 5$\lambda_{+}$ and loss/duplication ratios from 0 to 1 with all other parameters kept the same as the default. Indelible (Fletcher and Yang 2009) was used to simulate gap-free nucleotide sequences along the gene trees using the GTR + Γ model (Tavaré 1986) with two different sequence lengths: 500 and 100 bp and FastTree2 (Price *et al.* 2010) was used to estimate gene trees. We also performed some experiments with varying duplication rates and ILS levels while keeping the loss rate equal to the duplication rate. For detailed simulation settings, we refer the reader to Zhang *et al.* (2020).

We also experimented on a dataset of 100 taxa simulated by Molloy and Warnow (2020) and also used by Zhang *et al.* (2020). The duplication rate was varied as $\{0,1,2,5\}\times 10^{-10}$, and the loss rate was kept the same as the duplication rate. The ILS was controlled by varying the haploid population size as $\{1,5\}\times 10^{7}$, resulting in a much lower ILS level, 2% and 12%, respectively. Gene trees were estimated considering sequence lengths of 25, 50, 100, and 250 base pairs. We only considered the model conditions with 500 genes.

#### 5.1.2 Empirical dataset

We evaluated our method on three popular biological datasets. First, we use the transcriptome dataset of land plant species (Plants83), which was first analyzed by Wickett *et al.* (2014). We also reanalyzed a dataset of 16 yeast species (Fungi16) with 7280 multi-copy gene trees from Butler *et al.* (2009) and a dataset of 60 fungal species (Fungi60) with 5659 multi-copy gene trees from Morel *et al.* (2022).

#### 5.1.3 Methods and measurements

We evaluated different variants of DISCO paired with ASTRAL and wQFM, two leading species tree estimation methods. We also compared wQFM-DISCO (wQFM paired with DISCO) with ASTRAL-Pro, ASTRAL-DISCO, DupTree, and MulRF. Some other algorithms developed for multi-copy gene trees, such as iGTP (Chaudhary *et al.* 2010) and DynaDup variants (Bayzid *et al.* 2013, Saha *et al.* 2024), were excluded from consideration as previous studies have demonstrated their performance on the same dataset analyzed in this study. DynaDup-D, a variant of DynaDup that considers only duplication events, addresses the same problem as DupTree, and both methods demonstrate similar performance (Saha *et al.* 2024). iGTP and DynaDup-DL, which account for both duplications and losses, were found to be comparable to or worse than that of ASTRAL-Pro, DupTree, and DynaDup-D (Zhang *et al.* 2020, Saha *et al.* 2024). We ran ASTRAL-Pro 2, the memory-efficient version of ASTRAL-Pro in this experiment (Zhang and Mirarab 2022) and referred it as ASTRAL-Pro in the remainder of the article.

We compared the estimated trees (on simulated datasets) with the model species tree using normalized Robinson–Foulds (RF) distance (Robinson and Foulds 1981) to measure the tree error. The RF distance between two trees is the sum of the bipartitions (splits) induced by one tree but not by the other, and *vice versa*. For the biological dataset, we compared the estimated species trees to the scientific literature. We assessed support values in the estimated trees using local posterior probabilities (Sayyari and Mirarab 2016) computed by ASTRAL. The local posterior probabilities are calculated based on a transformation of normalized quartet scores (the percentage of quartets in the gene trees that agree with a branch in the estimated species tree). We analyzed multiple replicates of data for various model conditions. We performed the two-sided Wilcoxon signed-rank test, a non-parametric statistical test used to compare samples from two groups with the null hypothesis that the two groups come from the same distribution (Wilcoxon 1945). The statistical significance reported refers to the ranking of methods, rather than the absolute performance difference between them. We set the threshold α=0.05 to measure the statistical significance of the differences between the two methods.

## 6 Results and discussion

### 6.1 Experiment 1: evaluation of DISCO variants

In Experiment 1, we designed two separate experiments to assess the impact of different pruning strategies (pruning larger/smaller/randomly chosen subtree at a duplication node) and different rooting and tagging strategies (DISCO/DISCO-R with varying weights on duplication and loss). From the results presented in Section S3 of the Supplementary Material, we concluded that DISCO-R, along with equal weights for duplication and loss events, is the superior variant and used that in subsequent experiments.

### 6.2 Experiment 2: comparisons of best existing summary methods

In this experiment, we compared the best existing method ASTRAL-Pro with wQFM and ASTRAL paired with the best DISCO variant (DISCO-R) identified in our previous experiments. We also included DupTree and MulRF in our comparisons.

#### 6.2.1 Varying duplication and loss ratios

**Figure 2.**
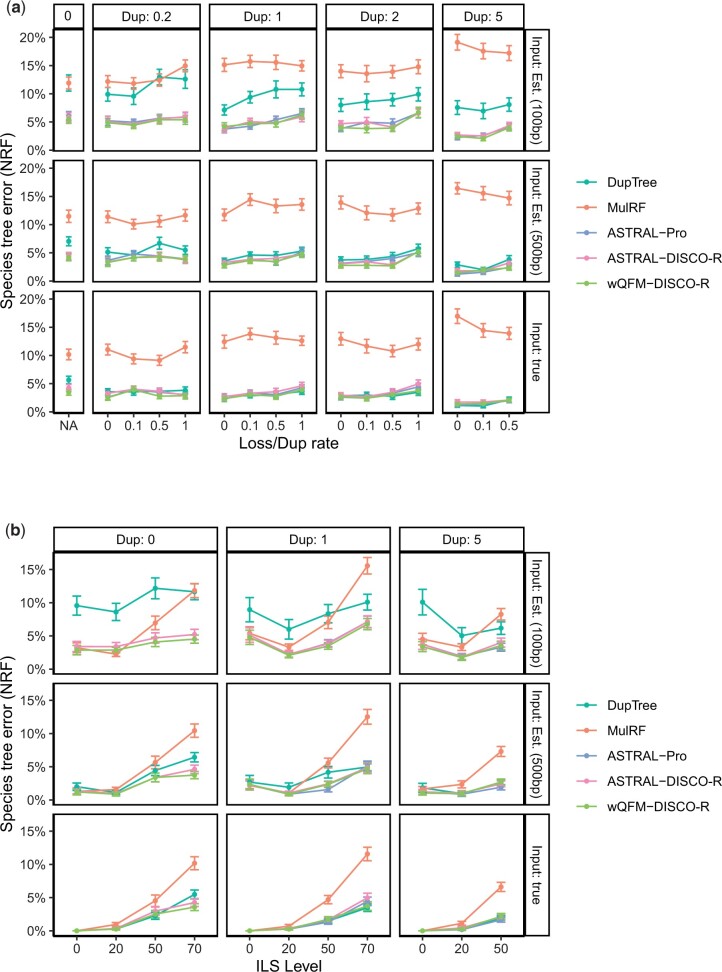
Comparison of wQFM-DISCO-R, ASTRAL-DISCO-R and some other existing methods on the S25 dataset with 1000 gene trees. We show the average RF rates with standard error bars over 50 replicates. Results for both true gene trees (simulated under the DLcoal method) and gene trees estimated by FastTree 2 from 100 and 500 bp alignments are presented. (a) Results when the duplication rate and loss/duplication ratio were varied. (b) Results when the duplication ratio and ILS level were varied.

First, we analyzed the 25-taxon dataset, where duplication rate and loss/duplication ratio were varied with ILS level fixed at 70% (see Fig. 2). On true gene trees, in seven of the 16 model conditions with varying duplication and loss rates, DupTree turns out to be the best method. However, in seven other conditions, DupTree performed worse than all other methods, except MulRF. However, wQFM-DISCO-R performed consistently well and it was better than other methods on average. The difference is statistically significant for all methods (*P *<* *.05), except ASTRAL-Pro. MulRF has notably higher errors (P≪.01) than the other methods across all model conditions.

On the estimated gene trees, the accuracy of DupTree dramatically decreased compared to its performance on the true gene trees. MulRF remained the worst method. wQFM-DISCO-R, ASTRAL-Pro, and ASTRAL-DISCO-R remained much more accurate than DupTree and MulRF. On aggregate metrics, such as mean or median, wQFM-DISCO-R performs better than ASTRAL-Pro and ASTRAL-DISCO-R in the majority of test conditions. In either setting of estimated gene trees (with 100 bp/500 bp), when all 800 data points (50 replicates each from 16 model conditions) were considered together, the superior performance of wQFM-DISCO-R over both ASTRAL-Pro and ASTRAL-DISCO-R is statistically significant (*P *<* *.05). In all 1600 points combined, the superiority of wQFM-DISCO-R over the other two is highly statistically significant (P≪.01).

Of the 16 combinations of duplication and loss rates under consideration, wQFM-DISCO-R turned out to be the best method 10, 10, 5 times and the second-best method 4, 5, 8 times for estimated trees with 100 bp genes, estimated trees with 500 bp genes, and true gene trees respectively. On the other hand, ASTRAL-Pro only came out to be the best method 4, 4, 4 times and the second-best method 4, 5, 7 times for estimated trees with 100 bp genes, estimated trees with 500 bp genes, and true gene trees, respectively. In all three input types, wQFM-DISCO-R is better on average than the other four methods in consideration.

#### 6.2.2 Varying ILS levels

We then repeat this experiment where ILS level also varies with the duplication rate, where loss/duplication rate is kept as 1. MulRF performs very well in conditions with no ILS, but as we increase the level of ILS, the performance of MulRF degrades drastically. wQFM-DISCO-R, ASTRAL-Pro, and ASTRAL-DISCO-R are relatively robust and much more tolerant of the level of ILS. Remarkably, similar to previous experiments, wQFM-DISCO-R was the best method. Of the 11 model conditions under consideration, wQFM-DISCO-R turned out to be the best method 9, 6, 6 times and the second-best method 2, 2, 2 times for estimated trees with 100 bp genes, estimated trees with 500 bp genes, and true gene trees. wQFM-DISCO-R is either significantly better (*P *<* *.05) than the other four methods, or there is no statistically significant difference between them.

Overall, under a wide range of model conditions with varying duplication/loss and ILS levels as well as varying gene tree estimation errors, wQFM paired with DISCO-R obtained the best performance. Out of a total 1650 test cases, it performed as good as or better than ASTRAL-Pro in 1503, and the improvement is statistically significant (*P *<* *.05).

#### 6.2.3 Varying parameters on the 100 taxa dataset

**Figure 3.**
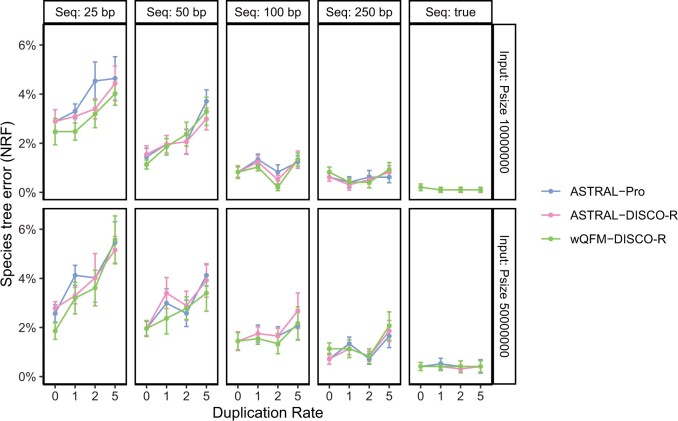
Comparison of wQFM-DISCO-R, ASTRAL-DISCO-R, ASTRAL-Pro on the S100 dataset with 1000 gene trees. We vary the duplication rate (box columns) and the loss rate (*x*-axis; the ratio of the loss rate to duplication rate). Results for both true gene trees and estimated gene trees from 25, 50, 100, and 250 bp alignments are presented. The effective haploid population size (labeled as Psize in the vertical axis) is also varied.

We then examined the effect of varying gene tree estimation error (controlled by the sequence length), ILS level (controlled by haploid population size), and duplication rate (with duplication/loss ratio kept to 1) in the 100 taxa dataset described in Subsection. ASTRAL-Pro was reported to be the superior method on this dataset (Zhang *et al.* 2020), especially when compared to DupTree and MulRF. Consequentially, we report DupTree and MulRF performance in our analysis. As we observe from Fig. 3, wQFM-DISCO-R turned out to be the best method in 25 out of the 40 model conditions and the second-best method in eight out of the 15 remaining conditions. Especially when the error is high (the case with 25 bp gene length), wQFM-DISCO-R came to be the best method in seven out of 8 cases.

Overall, in 29 model conditions, wQFM-DISCO-R performs as good as ASTRAL-Pro or better, and the improvement of wQFM-DISCO-R over ASTRAL is statistically significant (P≪.01).

Thus, wQFM is not only superior to ASTRAL on single-copy gene trees (as evident from previous studies (Mahbub *et al.* 2021, 2022)), wQFM paired with DISCO-R is more accurate and robust than ASTRAL-Pro under a wide variety of challenging model conditions.

### 6.3 Results on biological dataset

We present the results on the 1 kp plant dataset here (Wickett *et al.* 2014). Please see Section S4 in the Supplementary Material for results on fungal datasets.

#### 6.3.1 Plant (1KP) dataset

**Figure 4.**
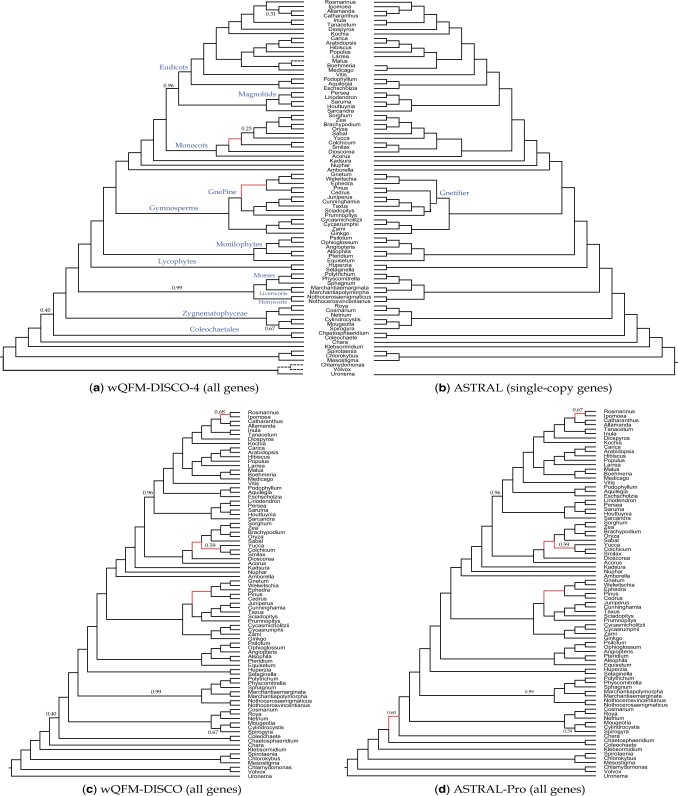
Analysis of the Plants83 dataset. (a) The tree obtained by wQFM-DISCO-R using all gene trees. Three genomes (indicated by dashed lines) were part of the multi-copy dataset but were absent from the single-copy data. (b) The tree reported in Wickett *et al.* (2014), estimated by ASTRAL using only the single-copy gene trees. The single-copy tree includes 23 species that were not in the multi-copy data and have been removed from the species tree. (c, d) Trees obtained by wQFM-DISCO and ASTRAL-Pro using multi-copy gene trees, respectively. Branch support (BS) values were computed using quartet-based local posterior probabilities. All BS values are 100% except where noted.

We reanalyzed the transcriptome dataset consisting of 103 plant species, which was initially analyzed by Wickett *et al.* (2014) using 424 single-copy gene trees with the ASTRAL method. The original study inferred 9683 multi-copy gene trees as well, containing up to 2395 leaves for 80 out of the 103 species and three additional genomes, making a total of 83 genomes. However, these multi-copy gene trees were not analyzed due to a lack of suitable methods. Later, ASTRAL-Pro analyzed these multi-copy gene trees. We reanalyzed this dataset using wQFM paired with both DISCO and DISCO-R. DISCO and DISCO-R decompose these multi-copy gene trees into 55 297 single-copy gene trees.wQFM-DISCO-R returned a tree that is highly congruent with the ASTRAL-Pro tree, differing only on three branches with low support (Fig. 4). wQFM-DISCO-R and ASTRAL-Pro trees differ from the ASTRAL tree based on single-copy gene trees reported by Wickett *et al.* (2014) in two and five branches, respectively. Both wQFM-DISCO-R and ASTRAL-Pro exhibit a significant difference from ASTRAL in that they both strongly support the GnePine hypothesis, where Gnetales is sister to Pinaceae, nested within the Coniferales. ASTRAL, on the other hand, recovered the Gnetifier hypothesis (i.e., Gnetales is sister to Coniferales as a whole). ASTRAL-Pro differs from both the ASTRAL and the concatenated analysis using super matrix in the placement of Yucca. In contrast, the placement of Yucca in the wQFM-DISCO-R-estimated tree aligns with ASTRAL and the concatenated analysis. Similarly, ASTRAL-Pro, unlike wQFM-DISCO-R, differs from both ASTRAL and concatenated analysis in the relative position of *Coleochaetale* and *Chara*. ASTRAL-Pro placed Chara and embryophytes as sisters which was rejected by most of the analyses in Wickett *et al.* (2014) (see Fig. 4 in Wicket *et al.*). Another discordance with low support is related to the placements of Rosmarinus and Ipomoea, where ASTRAL-Pro, unlike ASTRAL, concatenated analysis and wQFM-DISCO-R, grouped them together as sisters.

Comparing wQFM-DISCO-R and wQFM-DISCO, we found differences in two branches: (1) the placement of Yucca and (2) the sister relationships of Rosmarinus and Ipomoea. Concerning these two differences, wQFM-DISCO is aligned with ASTRAL-Pro, while wQFM-DISCO-R is aligned with the original ASTRAL and concatenated analysis. wQFM-DISCO tree, which exhibits discordance with the ASTRAL tree on four branches, is highly aligned with the ASTRAL-Pro tree, with only one difference pertaining to the placement of chara. Thus, all the methods inferred reasonable species trees, differing from each other in two to five branches (often with low support), where the ground truth is subject to debate.

### 6.4 Running time

The running time of wQFM is primarily dominated by its $\Omega (|\mathrm{leaf}{|}^{4})$ quartet generation step from gene trees. When the only cause of gene tree discordance is ILS, the number of leaves equals the number of taxa. However, with GDL in consideration, especially in cases where the duplication rate is much larger than the loss rate, the number of leaves is often many factors larger than the number of taxa. Running DISCO or DISCO-R ensures that the quartet generation step can run in $\Omega (\min{\left(|\mathrm{leaf}|,|\mathrm{taxon}|\right)}^{4})$ time. For analyzing the S100 dataset, the quartet generation took 4 h on average, but wQFM took only 10 min on average to infer a species tree from the weighted quartets. ASTRAL-Pro and ASTRAL-DISCO/ASTRAL-DISCO-R are faster and take less than 2 min on average to infer the species tree from gene trees. Generating the quartets from gene trees on the 1KP plant dataset with 83 species took seven hours, and running wQFM on the quartets took only 5 min. ASTRAL-Pro took 39 min to infer a species tree. The superior accuracy produced by wQFM further motivates us to focus on improving its running time. Especially, if wQFM could be run without explicitly enumerating all quartets from input gene trees (which is the most time-consuming step), it would be much more scalable to large datasets.

## 7 Conclusions

We introduced wQFM-DISCO as a remarkably accurate method for estimating species trees in the presence of gene duplication and loss events. The superior performance of wQFM-DISCO over ASTRAL-Pro across diverse model conditions demonstrates its efficacy and advancement in species tree estimation by effectively modeling both orthology and paralogy.

ASTRAL-Pro and DISCO are recent advancements in this field. ASTRAL-Pro, a recent summary method, is capable of modeling both orthology and paralogy. DISCO, a simple yet valuable strategy, enables the utilization of methods designed for single-copy gene trees on multi-copy gene trees. Our study examined various underlying hypotheses of DISCO and established its robustness by exploring different pruning strategies and scoring schemes for duplication and loss events. We proposed a novel pruning strategy in DISCO, referred to as DISCO-R, which enhances its accuracy and robustness by effectively incorporating more speciation-driven quartets. Finally, we proposed wQFM-DISCO as an adaptation of wQFM for estimating species trees despite the presence of paralogy. Extensive evaluations on diverse simulated and real datasets demonstrated that wQFM-DISCO consistently outperforms ASTRAL-Pro and other competing methods in terms of tree accuracy. Thus, wQFM-DISCO is sufficiently fast to analyze the datasets from many important phylogenomic studies (Wickett *et al.* 2014, Morel *et al.* 2022). Therefore, wQFM paired with DISCO represents a highly accurate and scalable method to account for both orthology and paralogy, which we believe should be considered as a potential tool to estimate species trees in the presence of ILS and GDL.

## Supplementary Material

vbae189_Supplementary_Data

## Data Availability

DISCO-R and other variants are freely available in open source form at https://github.com/skhakim/DISCO-variants. All the datasets analyzed in this article are from previously published studies and are publicly available.

## References

[vbae189-B1] Avni E , Cohen R, Snir S et al Weighted quartets phylogenetics. Syst Biol 2015;64:233–42. 25414175 10.1093/sysbio/syu087

[vbae189-B2] Bayzid MS. Inferring optimal species trees in the presence of gene duplication and loss: beyond rooted gene trees. J Comput Biol 2023;30:161–75. 36251762 10.1089/cmb.2021.0522

[vbae189-B3] Bayzid MS , Mirarab S, Warnow T. Inferring optimal species trees under gene duplication and loss. Proc. Pac Symp Biocomput (PSB) 2013;18:250–61. 10.1142/9789814447973_0025 23424130

[vbae189-B4] Bayzid MS , Warnow T. Estimating optimal species trees from incomplete gene trees under deep coalescence. J Comput Biol 2012;19:591–605. 22697236 10.1089/cmb.2012.0037

[vbae189-B5] Bayzid MS , Warnow T. Gene tree parsimony for incomplete gene trees: addressing true biological loss. Algorithms Mol Biol 2018;13:1. 29387142 10.1186/s13015-017-0120-1 PMC5774205

[vbae189-B6] Boussau B , Szöllosi GJ, Duret L et al Genome-scale coestimation of species and gene trees. Genome Res 2013;23:323–30. 23132911 10.1101/gr.141978.112 PMC3561873

[vbae189-B7] Butler G , Rasmussen MD, Lin MF et al Evolution of pathogenicity and sexual reproduction in eight Candida genomes. Nature 2009;459:657–62. 19465905 10.1038/nature08064 PMC2834264

[vbae189-B8] Chaudhary R , Bansal MS, Wehe A et al iGTP: a software package for large-scale gene tree parsimony analysis. BMC Bioinformatics 2010;11:574. 21092314 10.1186/1471-2105-11-574 PMC3002902

[vbae189-B9] Chaudhary R , Burleigh JG, Fernández-Baca D. Inferring species trees from incongruent multi-copy gene trees using the Robinson–Foulds distance. Algorithms Mol Biol 2013;8:1–12. 24180377 10.1186/1748-7188-8-28 PMC3874668

[vbae189-B10] Chifman J , Kubatko L. Quartet from SNP data under the coalescent model. Bioinformatics 2014;30:3317–24. 25104814 10.1093/bioinformatics/btu530 PMC4296144

[vbae189-B11] De Oliveira Martins L , Mallo D, Posada D. A bayesian supertree model for genome-wide species tree reconstruction. Syst Biol 2016;65:397–416. 25281847 10.1093/sysbio/syu082 PMC4851173

[vbae189-B12] Dunn CW , Howison M, Zapata F. Agalma: an automated phylogenomics workflow. BMC Bioinformatics 2013;14:330–9. 24252138 10.1186/1471-2105-14-330 PMC3840672

[vbae189-B13] Fitch WM. Homology: a personal view on some of the problems. Trends Genet 2000;16:227–31. 10782117 10.1016/s0168-9525(00)02005-9

[vbae189-B14] Fletcher W , Yang Z. INDELible: a flexible simulator of biological sequence evolution. Mol Biol Evol 2009;26:1879–88. 19423664 10.1093/molbev/msp098 PMC2712615

[vbae189-B15] Hejnol A , Obst M, Stamatakis A et al Assessing the root of bilaterian animals with scalable phylogenomic methods. Proc Biol Sci 2009;276:4261–70. 19759036 10.1098/rspb.2009.0896 PMC2817096

[vbae189-B16] Heled J , Drummond AJ. Bayesian inference of species trees from multilocus data. Mol Biol Evol 2010;27:570–80. 19906793 10.1093/molbev/msp274 PMC2822290

[vbae189-B17] Islam M , Sarker K, Das T et al STELAR: a statistically consistent coalescent-based species tree estimation method by maximizing triplet consistency. BMC Genomics 2020;21:136. 32039704 10.1186/s12864-020-6519-y PMC7011378

[vbae189-B18] Legried B , Molloy EK, Warnow T et al Polynomial-time statistical estimation of species trees under gene duplication and loss. J Comput Biol 2021;28:452–68. 33325781 10.1089/cmb.2020.0424

[vbae189-B19] Liu L. BEST: Bayesian estimation of species trees under the coalescent model. Bioinformatics 2008;24:2542–3. 18799483 10.1093/bioinformatics/btn484

[vbae189-B20] Mahbub M , Wahab Z, Reaz R et al wQFM: highly accurate genome-scale species tree estimation from weighted quartets. Bioinformatics 2021;37:3734–43. 34086858 10.1093/bioinformatics/btab428

[vbae189-B21] Mahbub S , Sawmya S, Saha A et al Quartet based gene tree imputation using deep learning improves phylogenomic analyses despite missing data. J Comput Biol 2022;29:1156–72. 36048555 10.1089/cmb.2022.0212

[vbae189-B22] Markin A , Eulenstein O. Quartet-based inference is statistically consistent under the unified duplication-loss-coalescence model. Bioinformatics 2021;37:4064–74. 34048529 10.1093/bioinformatics/btab414 PMC9113308

[vbae189-B23] Mim SA , Zarif-Ul-Alam M, Reaz R et al Quartet Fiduccia–Mattheyses revisited for larger phylogenetic studies. Bioinformatics 2023;39:btad332. 37285316 10.1093/bioinformatics/btad332 PMC10260390

[vbae189-B24] Mirarab S , Reaz R, Bayzid MS et al ASTRAL: genome-scale coalescent-based species tree estimation. Bioinformatics 2014;30:i541–8. 25161245 10.1093/bioinformatics/btu462 PMC4147915

[vbae189-B25] Molloy EK , Warnow T. FastMulRFS: fast and accurate species tree estimation under generic gene duplication and loss models. Bioinformatics 2020;36:i57–65. 32657396 10.1093/bioinformatics/btaa444 PMC7355287

[vbae189-B26] Morel B , Schade P, Lutteropp S et al Speciesrax: a tool for maximum likelihood species tree inference from gene family trees under duplication, transfer, and loss. Mol Biol Evol 2022;39:msab365. 35021210 10.1093/molbev/msab365 PMC8826479

[vbae189-B27] Price MN , Dehal PS, Arkin AP. Fasttree 2—approximately maximum-likelihood trees for large alignments. PLoS One 2010;5:e9490. 20224823 10.1371/journal.pone.0009490 PMC2835736

[vbae189-B28] Rasmussen MD , Kellis M. Unified modeling of gene duplication, loss, and coalescence using a locus tree. Genome Res 2012;22:755–65. 22271778 10.1101/gr.123901.111 PMC3317157

[vbae189-B29] Reaz R , Bayzid MS, Rahman MS. Accurate phylogenetic tree reconstruction from quartets: a heuristic approach. PLoS One 2014;9:e104008. 25117474 10.1371/journal.pone.0104008 PMC4130513

[vbae189-B30] Robinson D , Foulds L. Comparison of phylogenetic trees. Math. Biosci 1981;53:131–47.

[vbae189-B31] Saha P , Islam MS, Rahman T et al Gene tree parsimony in the presence of gene duplication, loss, and incomplete lineage sorting. In: *Proceedings of the Comparative Genomics: 21st International Conference, RECOMB-CG 2024, Boston, MA, USA, April 27–28, 2024*. Berlin, Heidelberg: Springer-Verlag, 2024, 110–28.

[vbae189-B32] Sayyari E , Mirarab S. Fast coalescent-based computation of local branch support from quartet frequencies. Mol Biol Evol 2016;33:1654–68. 27189547 10.1093/molbev/msw079 PMC4915361

[vbae189-B33] Snir S , Rao S. Quartets MaxCut: a divide and conquer quartets algorithm. IEEE/ACM Trans Comput Biol Bioinform 2010;7:704–18. 21030737 10.1109/TCBB.2008.133

[vbae189-B34] Szöllősi GJ , Tannier E, Daubin V et al The inference of gene trees with species trees. Syst Biol 2015;64:e42–62s. 25070970 10.1093/sysbio/syu048 PMC4265139

[vbae189-B35] Tavaré S. Some probabilistic and statistical problems in the analysis of DNA sequences. Lect Math Life Sci 1986;17:57–86.

[vbae189-B36] Warnow T. 2017. Computational Phylogenetics: An Introduction to Designing Methods for Phylogeny Estimation. Cambridge, England: Cambridge University Press.

[vbae189-B37] Wehe A , Bansal MS, Burleigh JG et al DupTree: a program for large-scale phylogenetic analyses using gene tree parsimony. Am J Bot 2008;24:1540–1. 10.1093/bioinformatics/btn230 18474508

[vbae189-B38] Wickett NJ , Mirarab S, Nguyen N et al Phylotranscriptomic analysis of the origin and early diversification of land plants. Proc Natl Acad Sci U S A 2014;111:E4859–68. 25355905 10.1073/pnas.1323926111 PMC4234587

[vbae189-B39] Wilcoxon F. Individual comparisons by ranking methods. Biometrics Bull 1945;1:80–3. 18903631

[vbae189-B40] Willson J , Roddur MS, Liu B et al DISCO: species tree inference using multicopy gene family tree decomposition. Syst Biol 2022;71:610–29. 34450658 10.1093/sysbio/syab070 PMC9016570

[vbae189-B41] Yang Y , Smith SA. Orthology inference in nonmodel organisms using transcriptomes and low-coverage genomes: improving accuracy and matrix occupancy for phylogenomics. Mol Biol Evol 2014;31:3081–92. 25158799 10.1093/molbev/msu245 PMC4209138

[vbae189-B42] Zhang C , Mirarab S. ASTRAL-Pro 2: ultrafast species tree reconstruction from multi-copy gene family trees. Bioinformatics 2022;38:4949–50. 36094339 10.1093/bioinformatics/btac620

[vbae189-B43] Zhang C , Scornavacca C, Molloy EK et al ASTRAL-Pro: quartet-based species-tree inference despite paralogy. Mol Biol Evol 2020;37:3292–307. 32886770 10.1093/molbev/msaa139 PMC7751180

[vbae189-B44] Zhang L. From gene trees to species trees II: species tree inference by minimizing deep coalescence events. IEEE/ACM Trans Comput Biol Bioinform 2011;8:1685–91. 21576759 10.1109/TCBB.2011.83

