# Supplementary material for: wQFM-DISCO: DISCO-enabled wQFM improves phylogenomic analyses despite the presence of paralogs
Source: Bioinform Adv. 2024 Nov 27;4(1):vbae189. doi: 10.1093/bioadv/vbae189 (PMC11634537; doi:10.1093/bioadv/vbae189)
Supplement: vbae189_Supplementary_Data [file vbae189_supplementary_data.pdf]

Supplementary Material to wQFM-DISCO: DISCO-enabled wQFM improves phylogenomic analyses despite the presence of paralogs

Sheikh Azizul Hakim[1][0000−0002−3405−2402], Md. Rownok Zahan Ratul[1][0009−0004−8263−0993], and Md. Shamsuzzoha Bayzid[1,*][0000−0002−5640−0615]

Department of Computer Science and Engineering,
Bangladesh University of Engineering and Technology,
Dhaka-1205, Bangladesh
{hakim, 1805019, shams_bayzid}@cse.buet.ac.bd
*Corresponding Author

1 Background and preliminaries

We now briefly overview the DLCoal model, ASTRAL-Pro, DISCO, and wQFM to make this paper self-contained.

1.1 DLCoal model

The following brief overview of the unified duplication-loss-coalescence (DLCoal) model has been adapted from (11; 16).

A *species tree* represents the evolutionary history of a group of species. Its leaves are labeled by the extant species names. A *locus tree* represents a duplication-loss history of a particular gene. A locus tree is obtained from a species tree by running the duplication/loss process top-down along the edges of the species tree. In other words, the duplication/loss process is a birth-death process with a fixed birth (duplication) rate and death (loss) rate (1). The birth-death process starts in the root edge of the species tree; whenever it reaches a speciation point, the process splits into two copies and continues independently in the children edges. A locus tree node can be termed as *speciation* if such a node corresponds to a speciation event/node in the species tree or as *duplication* if such a node corresponds to creating a new locus. Note that locus tree leaves are labeled by gene names. The *gene tree* is obtained from a locus tree by running the bounded multi-species coalescent (b-MSC) process bottom-up along the edges of the locus tree. The duplication/loss process and

the b-MSC process are jointly referred to as the DLCoal model. In the standard multi-species coalescent (MSC) model, there is exactly one gene lineage starting in every extant locus tree leaf. If two or more lineages enter the same locus tree edge, then the coalescence history of these lineages is determined by an exponential distribution. The b-MSC process in the unified DLCoal model is defined by imposing constraints on MSC due to the duplication points. In particular, all lineages originating below a daughter duplicate must coalesce below the respective duplication node. Given a model of evolution, any species tree estimation method is *statistically consistent* under the specified model if the method is guaranteed to produce the true species tree when sufficiently many error-free gene trees are provided as input.

1.2 ASTRAL

ASTRAL (12) is a polynomial time coalescent-based species tree method that provides statistical consistency estimation of the true species trees from unrooted gene trees, under the multi-species coalescent (MSC) model. Given a set of unrooted single-copy gene trees and a set of bipartitions, ASTRAL finds the species tree that agrees with the largest number of quartet trees induced by the set of gene trees. Formally, ASTRAL solves the following $\mathcal{NP}$-hard problem using a dynamic programming based approach.

**Problem**	**Maximum Quartet Support Species Tree ($\mathcal{MQSST}$)**
INPUT	Set $\mathcal{T}$ of unrooted gene trees, each leaf-labelled by species set S, and set χ of bipartitions on S.
OUTPUT	Tree T on species set S that draws its bipartitions from χ such that $\sum_{q \in Q(T)} w(q, T)$ is maximized, where $Q(T)$ is the set of quartet trees induced by T and $w(q, \mathcal{T})$ is the number of the trees in $\mathcal{T}$ that induce quartet topology q.

When the input set of bipartitions (χ) is set to all bipartitions from the input set of unrooted gene trees, ASTRAL is a statistically consistent estimator under MSC. Subsequent versions of ASTRAL (ASTRAL-II (13), ASTRAL-III (22)) restricts the input set of bipartitions for computational efficiency without compromising the statistical consistency guarantees.

1.3 wQFM

wQFM considers the weighted version of the *Maximum Quartet Consistency* (MQC) problem (17; 19), which we call $\mathcal{WMQC}$, and define as follows.

**Problem**	**Weighted Maximum Quartet Consistency ($\mathcal{WMQC}$)**
INPUT	A set $\mathcal{Q}$ of weighted quartets on a set $\mathcal{S}$ of taxa.
OUTPUT	A tree T on $\mathcal{S}$ such that the total weight of the quartets in $\mathcal{Q}$ that are consistent with T is maximized.

wQFM, in general, can be used to amalgamate any set of given weighted quartets (e.g., quartets induced by the gene trees or the quartets produced by SVDquartets). In this study, we consider wQFM-GTF, which estimates species trees by combining the quartets induced by the gene trees, and the weights of the quartets are computed based on the gene tree frequency (GTF), i.e., we use the frequency of the quartets in input gene trees as weights. wQFM-GTF essentially solves the same $\mathcal{MQSST}$ problem as ASTRAL. Although ASTRAL uses an exact dynamic programming based approach, wQFM-GTF applies a heuristic approach (based on the FM algorithm (6; 17)). As such, theoretical analysis of wQFM-GTF is difficult, but wQFM-GTF has been shown to outperform ASTRAL in a number of prior studies (8; 9).

1.4 ASTRAL-Pro

ASTRAL-Pro is an extension of the ASTRAL-like approach when multi-copy gene family trees are applied as input. We begin this section by reviewing the terminology introduced in (23).

Let $\mathcal{S}$ be a set of n species. The input to our problem is a collection of multi-copy gene trees. Given a rooted multi-copy gene tree G, we define a many-to-one mapping α_G between its leaf-set $\mathcal{L}_G$ and $\mathcal{S}$. For a leaf $l \in \mathcal{L}_G$, $\alpha_G(l)$ denotes the species from which the gene l was taken. For a node u, we define $\mathcal{L}_G(u)$ as the set of leaves under u. We extend definitions such that $\alpha_G(A) = \{\alpha_G(a)|a \in A\}$ for $A \subset \mathcal{L}_G$ and $\alpha_G(u) = \alpha_G(\mathcal{L}_G(u))$ for a node u in G. In other words, $\alpha_G(u)$ denotes the set of species whose genes are present under a node u in G.

A quartet Q is a four-membered subset $\mathcal{L}_G$. If we recursively delete leaves from G such that only four leaves in Q are left, we are left with a tree with two degree-3 nodes. These two nodes are called the anchors of Q. Since G is rooted, we can find the lowest common ancestor (LCA) of these two nodes, referred to as the anchor LCA of Q in G.

We tag every non-leaf node as speciation or duplication using Algorithm 1. Given a non-leaf node u with two children u_1 and u_2, we tag u as a speciation node if $\alpha_G(u_1) \cap \alpha_G(u_2) = \{\}$. Otherwise, we tag u as a duplication node. A quartet with a topology $ab|cd$ in a gene tree is defined as a speciation-driven quartet (SQ) if all the four genes are contained in different species and the LCA of either a or b with either c or d is tagged as speciation. Two SQs on the same four species are equivalent if they have the same anchor LCA.

Algorithm 1 Tagging and Rooting heuristic used by ASTRAL-Pro (23). In default setting, duplication and loss weights are both set to 1.

```
procedure TAG_AND_ROOT(G)
    s ← −∞
    for edge e in G do
        root G at e and let r_e be the new root
        s_e ← TAG(r_e)
        if s_e < s then
            r ← r_e
            s ← s_e
        end if
    end for
    root at r
    TAG(r)
end procedure

procedure TAG(u)
    if u is a leaf then
        return 0
    end if
    u_l, u_r ← children of u
    score(u) ← TAG(u_l) + TAG(u_r)
    if α_G(u) and α_G(u_l) are disjoint then
        tag u as Speciation
    else
        tag u as Duplication
        if α_G(u) = α_G(u_l) ∪ α_G(u_r) then
            if α_G(u_l) = α_G(u_r) then
                score(u) ← score(u)+ duplication_weight
            else
                score(u) ← score(u)+ duplication_weight + loss_weight
            end if
        else
            score(u) ← score(u)+ duplication_weight +2 × loss_weight
        end if
    end if
    return score(u)
end procedure
```

An equivalent quartet class is a set of quartets equivalent with one another. The per-locus quartet score of a species tree S with respect to a rooted tagged gene tree G is the number of equivalent quartet classes that match the S topology. The *Maximum per-Locus Quartet score Species Tree* (MLQST) problem is to find the species tree that maximizes the per locus quartet score with respect to input gene trees, given a set of rooted tagged gene trees. ASTRAL-Pro, in its exact version, solves the MLQST problem using dynamic programming.

When the input gene trees are caused by MSC only, without any duplication/loss, every quartet can be considered as 'speciation-driven.' Hence, the MLQST problem reduces to the MQSST problem, which can be solved by ASTRAL (exactly) or wQFM (heuristically).

1.5 DISCO

The core concept of DISCO is to break down each multi-copy gene tree into a collection of single-copy gene trees (21), enabling methods designed for single-copy gene trees to be applied to datasets containing multi-copy gene trees. To accomplish this, DISCO uses Algorithm 1, the rooting and tagging algorithm used by ASTRAL-Pro. Algorithm 1 roots and tags using the maximum parsimony principle with equal penalties for duplication and loss, and no penalty for ILS. Given a fully resolved unrooted binary tree, Algorithm 1 places the root along an edge to minimize the total number of duplications and losses. After rooting the tree, each internal node is tagged either as a duplication or a speciation node. The workflow of DISCO is shown in Algorithm 2. Given a rooted tagged gene tree g, the set of output trees S_g is initially empty. The decomposition algorithm performs a postorder traversal. When it reaches a duplication node, the smaller subtree rooted at that node is pruned and added to S_g. When the entire traversal is complete, the resultant tree is also added to S_g. It can be ensured that every tree in S_g is a single-copy tree.

1.6 DupTree

The `DupTree` method is a maximum parsimony-based approach for inferring species trees from collections of gene trees, minimizing the number of gene duplications (20). In the context of gene tree parsimony (GTP), `DupTree` aims to find a species tree that achieves the minimum reconciliation cost, defined strictly in terms of gene duplications.

`DupTree` employs an efficient heuristic search based on rooted Subtree Pruning and Regrafting (rSPR) operations, enhancing computational performance over previous Gene Tree Parsimony (GTP) implementations like GeneTree(15). The algorithm iteratively refines species trees by evaluating alternative gene tree

Algorithm 2 The pseudocode of DISCO. (21)

Require: A rooted multi-copy gene tree g with each node tagged as duplication/speciation
Ensure: A set of single-copy gene trees S_g

```
S_g ← ∅
for node v in the postorder traversal of g do
    if v is a duplication node then
        g_{v_l}, g_{v_r} ← be the left and right subtrees of v
        g_v ← g_{v_l} if |leaves(g_{v_l})| > |leaves(g_{v_r})| else g_{v_r}
        delete g_v from g
        S_g ← S_g ∪ {g_v}
    end if
end for
S_g ← S_g ∪ {g}
```

rootings and recalculating reconciliation costs. It supports unrooted input gene trees and weighted GTP analyses, handling large-scale phylogenetic datasets effectively.

By minimizing duplication events, `DupTree` is well-suited for genome-wide phylogenetic analyses where gene tree discordance is a concern.

1.7 iGTP

`iGTP` is a method for addressing three principal variants of the gene tree parsimony (GTP) problem: (i) the duplication problem, which minimizes the number of gene duplications; (ii) the duplication-loss problem, which minimizes both gene duplications and losses; and (iii) the deep-coalescence problem, which minimizes the number of deep coalescence events (3). These problems seek to reconcile conflicts between gene trees and a species tree by minimizing the reconciliation cost, defined by the fewest evolutionary events required to explain gene tree discordance.

In the context of GTP, `iGTP` employs heuristic search algorithms to approximate solutions efficiently, as exact methods are computationally intractable for large datasets. The method iteratively refines species trees by minimizing reconciliation costs across input gene trees. Through this approach, `iGTP` supports large-scale phylogenetic analyses, enabling the inference of species trees from extensive sets of gene trees where discordance is prevalent. The flexibility of `iGTP` in handling different reconciliation cost metrics makes it well-suited for exploring complex evolutionary histories where duplication, loss, and deep coalescence events shape gene tree topologies.

1.8 MulRF

The `MulRF`(4) method extends the Robinson–Foulds (RF) distance to multi-labeled trees for inferring species trees from collections of multi-copy gene trees (4). Given a set of unrooted, multi-copy gene trees where multiple leaves may share the same label, `MulRF` aims to find a species tree that minimizes the sum of the generalized RF distances to the input trees. To compute this, `MulRF` first extends the species tree by adding additional copies of leaf labels in a star tree topology, allowing for calculating the RF distance between the multi-copy gene tree and the extended species tree.

The MulRF distance between a species tree and an input gene tree can be computed in linear time concerning the number of leaves in the multi-copy gene tree. The method defines a MulRF supertree as the species tree that minimizes the total MulRF distance across all input gene trees. As computing the optimal MulRF supertree is NP-hard, the method employs a heuristic based on unrooted Subtree Pruning and Regrafting (SPR) local search to efficiently estimate the species tree.

2 An illustrative example where DISCO misses a species altogether, but DISCO-R does not

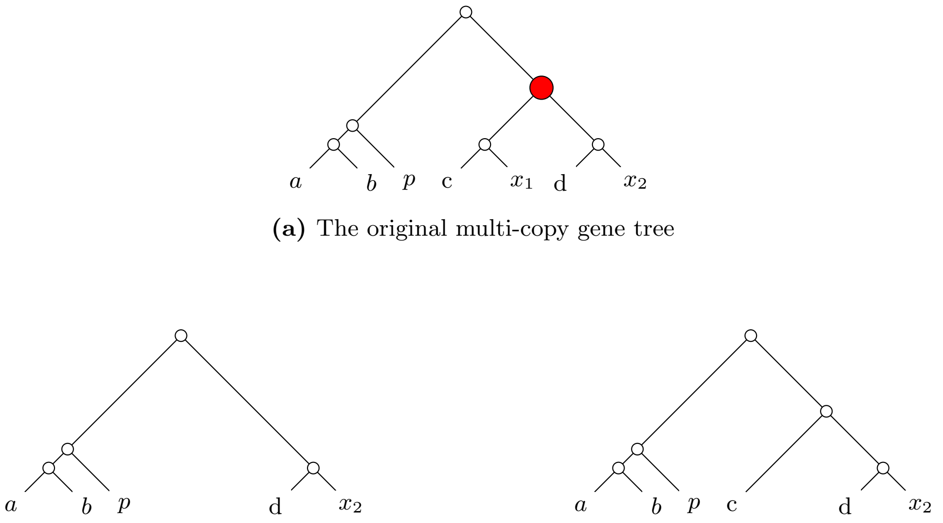

(a) The original multi-copy gene tree

(b) Decomposition output of DISCO

(c) Decomposition output of DISCO-R

Fig. 1: An example where DISCO misses a certain taxon (c) in its output. At the duplication node (marked as red), the subtree (c, x_1) is pruned, which does not induce any quartet and is left out of DISCO output. However, DISCO-R regrafts the node c back to the gene tree.

3 DISCO Variants

3.1 Variants of DISCO and DISCO-R used in experiments

DISCO uses the rooting and tagging heuristic shown in Algorithm 1, assigning equal weights to each duplication and loss event. Then, the smaller subtree is pruned at each duplication node while doing a postorder traversal. We wanted to examine the effects of various weighting schemes and pruning strategies. We experimented with several variants of DISCO (e.g., DISCO-1, DISCO-2, ..., DISCO-6). First, instead of pruning the smaller subtree at a duplication node as was done in the original DISCO algorithm (line 5 of Algorithm 2), we experimented with pruning the larger subtree (DISCO-2) or randomly pruning one of the two subtrees (DISCO-3) at that node. We also experimented with the rooting algorithm of ASTRAL-Pro, where we assigned the cost of a duplication event to be twice the cost of a loss event (DISCO-5,-6). Such scoring heuristic was previously suggested in literature (5; 23). Zheng *el al.* also hypothesized that Algorithm 1 (and hence ASTRAL-Pro) is somewhat robust on the underlying rooting and tagging heuristic. . Table 1 shows six different variants we explored in this study.

Table 1: Different variants of DISCO analyzed in this study, covering various pruning strategies and scoring schemes for duplication and loss events.

Variant	Pruning Strategy	Subtree to be pruned	Duplication Weight	Loss Weight
DISCO-1	DISCO	Smaller Subtree	1	1
DISCO-2	DISCO	Larger Subtree	1	1
DISCO-3	DISCO	Random Subtree	1	1
DISCO-4	DISCO-R	Smaller Subtree	1	1
DISCO-5	DISCO	Smaller Subtree	1	0.5
DISCO-6	DISCO-R	Smaller Subtree	1	0.5

3.2 Experiment 1: evaluation of DISCO variants

In Experiment 1, we have two separate experiments to assess: 1(a) the impact of different pruning strategies, and 1(b) the impact of different rooting and tagging strategies.

Experiment 1(a): Effects of choosing the subtree to be pruned We examined how the performance varies by pruning the smaller (DISCO-1), larger (DISCO-2), or one of them at random (DISCO-3). Originally,

DISCO prunes the smaller subtree at a duplication node, but no justification was provided for their choice (21). We compared these three methods both with ASTRAL and wQFM with varying duplication rates and loss/duplication ratios (see Figure 2). When the duplication rate is 0, the methods do not differ since the gene trees are single-copy and hence there is no pruning involved. Otherwise, among the 15 other model conditions, we see that ASTRAL-DISCO-1 performs better than both ASTRAL-DISCO-2 and ASTRAL-DISCO-3 on 12 model conditions with estimated gene trees with gene length of 100 bp, on 12 model conditions with estimated gene trees with gene length of 500 bp, and on 11 model conditions with true gene trees. Similarly, wQFM-DISCO-1 showed superior performance compared to wQFM-DISCO-2 and wQFM-DISCO-3 on 11, 11, and 12 model conditions, respectively, across these three types of gene trees. In each case, the difference is statistically significant ($p \ll 0.01$). ASTRAL and wQFM paired with DISCO-2 (i.e., pruning the larger trees) produced the worst results in most of the cases. Relative performances of different variants of DISCO paired with either ASTRAL or wQFM are similar. Thus, we experimentally conclude that pruning the smaller subtree is the better strategy. Pruning the smaller subtree retains more speciation-driven quartets, which could be a plausible explanation for our findings. Therefore, in subsequent experiments, we only prune the smaller subtrees as in the original DISCO algorithm.

Experiment 1(b): Effects of different weighting schemes in the tagging technique of ASTRAL-Pro and DISCO The tagging heuristic used by ASTRAL-Pro (and also by DISCO) equally weights each duplication and loss event. We examined the effects of assigning the weight of a duplication event two times that of a loss event. We also experimented with DISCO-R, the pruning technique we introduced in Section **??**. We compared these methods (DISCO-1,4,5,6) paired with both ASTRAL and wQFM under various model conditions (see Figure 3). The relative ranks of the methods are shown in Table 2. Figure 3 and Table 2 suggest that regardless of the choice of species tree estimation methods (i.e., ASTRAL or wQFM), DISCO-R with two different weighting schemes (i.e., DISCO-4 and DISCO-6) are frequently better than original DISCO variants (DISCO-1 and DISCO-5) and the improvements of DISCO-R variants over the original DISCO variants are sometimes statistically significant ($p < 0.05$). However, on true gene trees, all DISCO and DISCO-R variants achieved comparable accuracy, with DISCO variants having a slight advantage over DISCO-R variants on some of the model conditions. In nearly every model condition, DISCO-4 and DISCO-6 methods (paired with ASTRAL or wQFM) had very similar accuracy, with neither reliably having an advantage over the other. Similar observations hold for DISCO-1 and DISCO-5 (i.e. original DISCO variants with different weighting schemes). This indicates that the rooting and tagging heuristic incorporated by

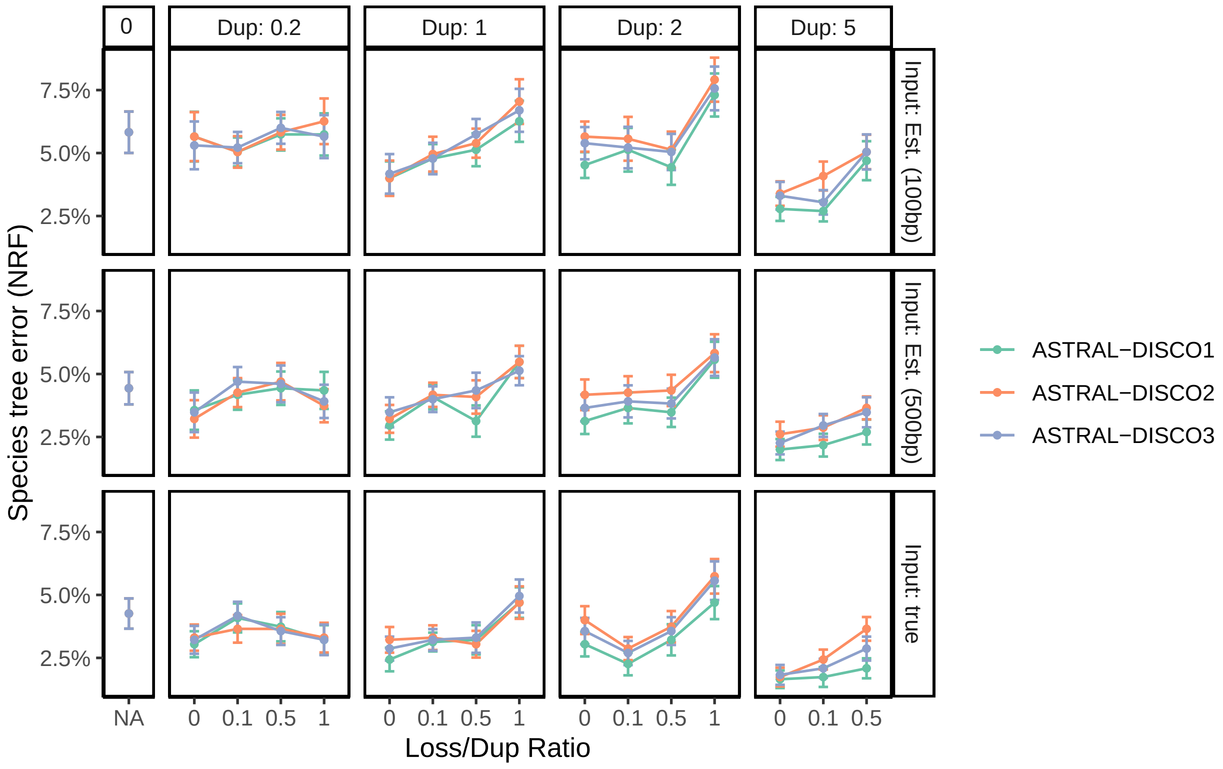

(a)

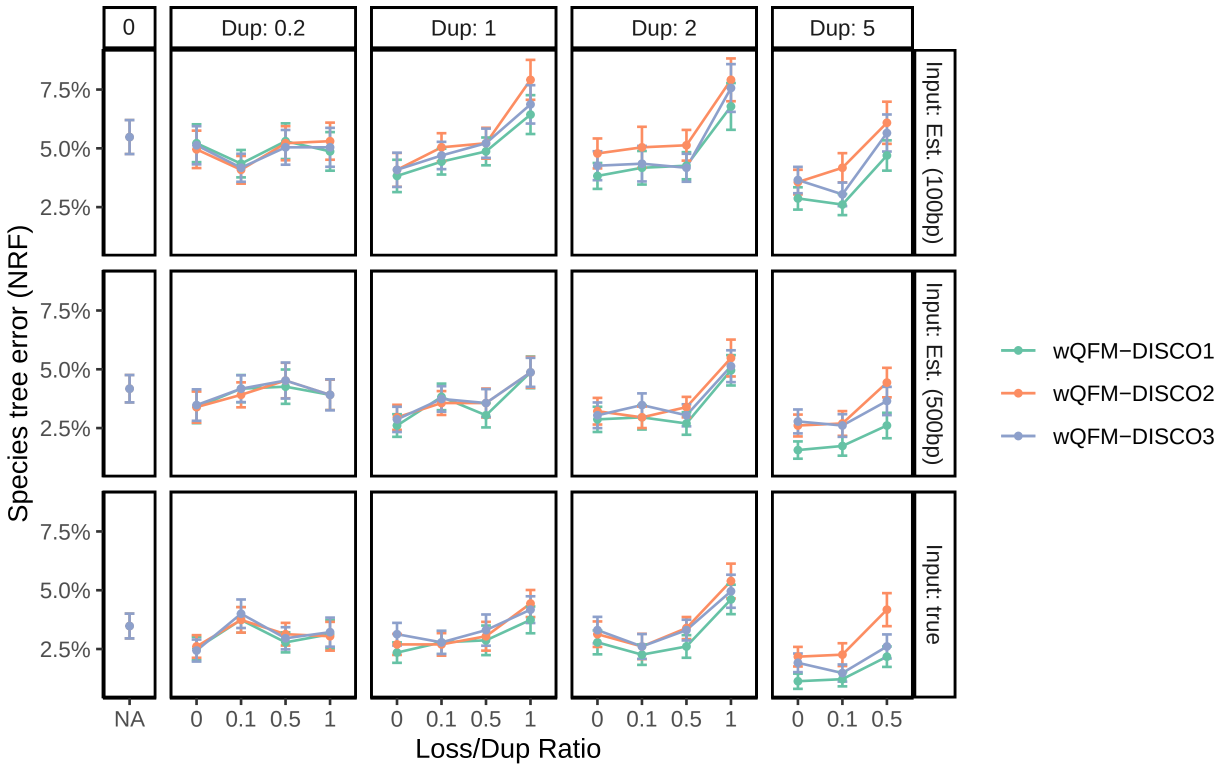

(b)

Fig. 2: Comparison of variants of DISCO with different pruning techniques, paired with both ASTRAL and wQFM, on the S25 dataset with 1000 gene trees. We show the average RF rates with standard error bars over 50 replicates. We vary the duplication rate (box columns) and the loss rate (x-axis; the ratio of the loss rate to duplication rate). Results for both true gene trees and estimated gene trees from 100 and 500 bp alignments are presented. (a) Results for ASTRAL, paired with different DISCO variants. (b) Results for wQFM paired with DISCO variants.

ASTRAL-Pro and also by DISCO is considerably robust to the assignments of weights to duplication and speciation events. These results indicate that our proposed DISCO-R provides more accurate and robust results than the original DISCO algorithm and supports our findings that DISCO-R retains more species-driven quartets than DISCO (as discussed in Section 3 of the original paper). Therefore, in subsequent experiments, we consider only DISCO-4 (DISCO-R with an equal weighting scheme).

We also observe that in each of the 48 model conditions and 6 variants of DISCO discussed, wQFM paired with any certain variant performed better than ASTRAL paired with that variant in 243 out of 288 times and this improvement is highly statistically significant ($p << 0.01$). This finding supports the previously obtained result that wQFM is experimentally superior to ASTRAL (8; 9).

Table 2: Effects of different weighting schemes in the tagging technique of ASTRAL-Pro and DISCO. We present how the four DISCO variants ranked when combined with both ASTRAL and wQFM are shown in various types of inputs. For example, out of the 15 model conditions with estimated gene trees with 500 bp genes, wQFM-DISCO4 ranked first seven times, second 3 times, and so on. In the case of a group of records with the same value, the lowest rank in the group was assigned.

Method	Rank			
	1st	2nd	3rd	4th
ASTRAL-DISCO1	1	2	8	4
ASTRAL-DISCO4	7	5	2	1
ASTRAL-DISCO5	3	0	8	4
ASTRAL-DISCO6	7	5	3	0

(a) Estimated (100bp)

Method	Rank			
	1st	2nd	3rd	4th
wQFM-DISCO1	5	1	7	2
wQFM-DISCO4	6	3	3	3
wQFM-DISCO5	5	1	6	3
wQFM-DISCO6	5	4	6	0

(b) Estimated (100bp)

Method	Rank			
	1st	2nd	3rd	4th
ASTRAL-DISCO1	4	2	5	4
ASTRAL-DISCO4	8	4	2	1
ASTRAL-DISCO5	4	2	8	1
ASTRAL-DISCO6	6	4	3	2

(c) Estimated (500bp)

Method	Rank			
	1st	2nd	3rd	4th
wQFM-DISCO1	5	2	7	1
wQFM-DISCO4	7	3	5	0
wQFM-DISCO5	5	3	7	0
wQFM-DISCO6	6	2	2	5

(d) Estimated (500bp)

Method	Rank			
	1st	2nd	3rd	4th
ASTRAL-DISCO1	7	2	5	1
ASTRAL-DISCO4	4	3	4	4
ASTRAL-DISCO5	7	3	3	2
ASTRAL-DISCO6	8	1	4	2

(e) True

Method	Rank			
	1st	2nd	3rd	4th
wQFM-DISCO1	8	1	5	1
wQFM-DISCO4	4	3	8	0
wQFM-DISCO5	6	3	4	2
wQFM-DISCO6	5	1	7	2

(f) True

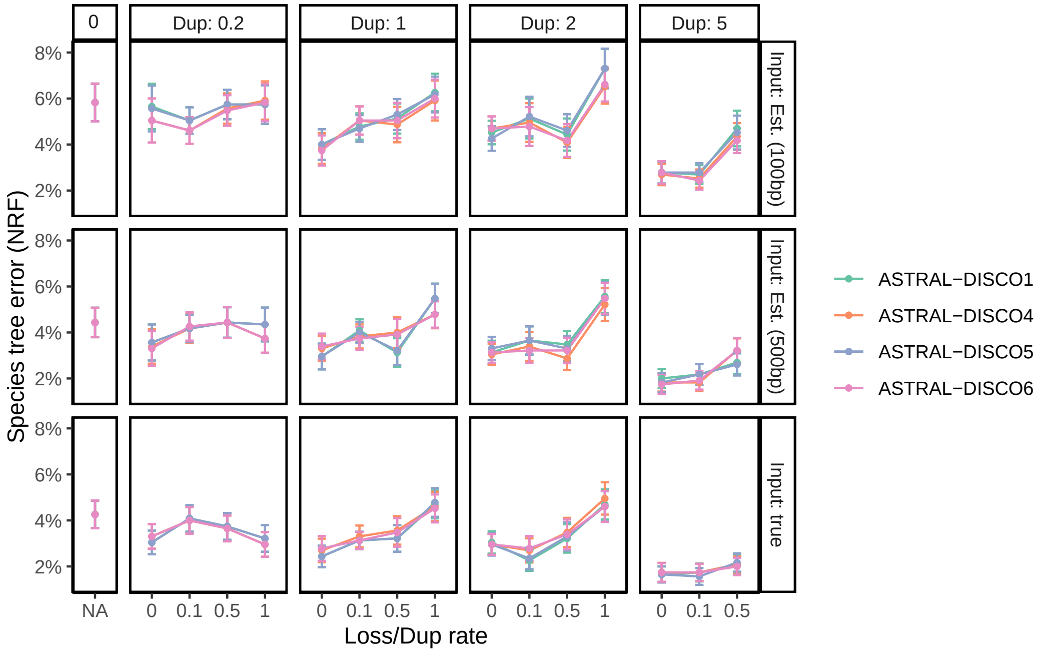

(a)

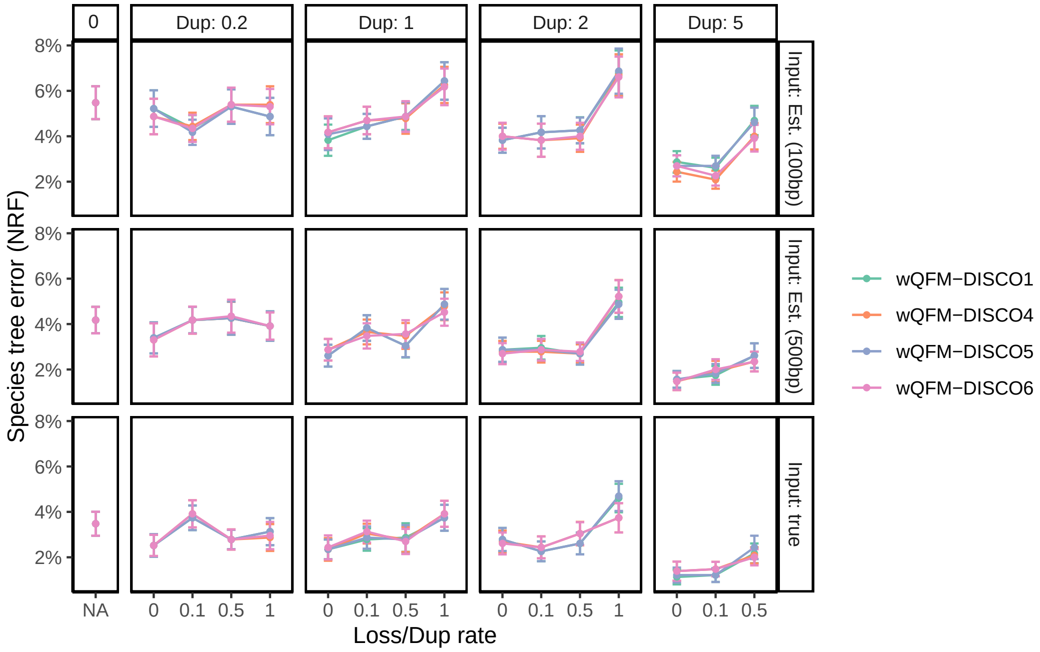

(b)

Fig. 3: Comparison of variants of DISCO (two different weighting schemes with two different pruning techniques, producing four DISCO variants), paired with both ASTRAL and wQFM, on the S25 dataset with 1000 gene trees. We show the average RF rates with standard error bars over 50 replicates. We vary the duplication rate (box columns) and the loss rate (x-axis; the ratio of the loss rate to duplication rate). Results for both true gene trees and estimated gene trees from 100 and 500 bp alignments are presented. (a) Results for ASTRAL, paired with different DISCO variants. (b) Results for wQFM paired with DISCO variants.

4 Analysis of Fungal Datasets

We analyzed two fungal datasets: 1) a data set containing 16 yeast species with 7,280 multi-copy gene families available from (2), and 2) a 60-species dataset from (14).

16-species yeast dataset. Butler et al. (2009) analyzed only single copy gene trees based on 706 one-to-one orthologs (2). We re-analyzed the data considering all the 7,280 multi-copy gene trees (see Fig. 4(b). wQFM-DISCO, wQFM-DISCO-4 and ASTRAL-Pro returned an identical tree, which differs from the tree reported in the original study on only one branch, introducing discordance in the relative position of *Saccharomyces castellii* and *Candida glabrata*. The original study placed *Saccharomyces castellii* as sister to *Candida glabrata* and the *Saccharomyces* group which was imposed by a constraint during the ML search. ASTRAL-Pro, wQFM-DISCO and wQFM-DISCO-4 trees placed *Candida glabrata* as sister to the *Saccharomyces* group, which is aligned with the unconstrained ML search in the concatenated analysis and the tree reported by Salichos and Rokas (18).

60-species fungal dataset. (14) extracted a fungal dataset of 60 species from PhylomeDB (7). In their study, they found that ASTRAL-Pro, SpeciesRAX and FastMulRFS infer the same tree. In our study, we found that wQFM-DISCO-4 infers the same tree as well, which agrees with the species tree obtained by concatenation (10) (see Fig. 4(a). However, this tree contradicts the relationship established in the literature regarding one specific branch, related to the positioning of the clade comprising Chytridiomycota (*Batrachochytrium dendrobatidis*) and Zygomycota (*Rhizopus oryzae* and *Phycomyces blakesleeanus*). (14) discussed the biological phenomenon of this split in detail.

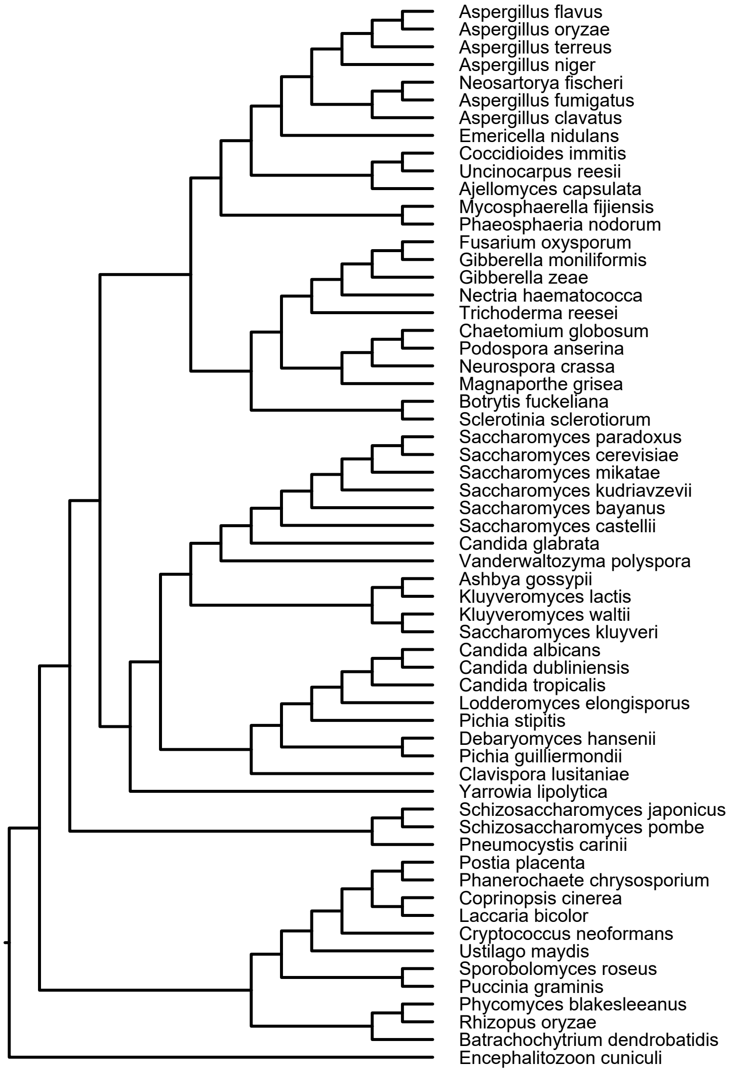

(a) Fungi60

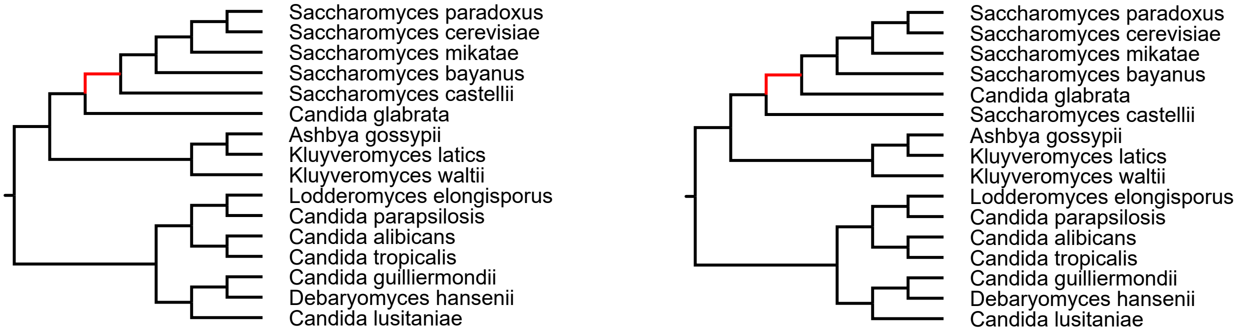

wQFM-DISCO-4, ASTRAL-Pro

Concatenation (single-copy genes)

(b) Fungi16

Fig. 4: Analysis of the Fungi16 and Fungi60 datasets. (a) Fungi60 dataset: the species tree returned by both wQFM-DISCO-4 and ASTRAL-Pro using all genes. (b) Fungi16 dataset. Left: the species tree returned by both wQFM-DISCO-4 and ASTRAL-pro. Right: the tree obtained by concatenation of 706 single-copy genes with the red branch enforced as a constraint (2).

Bibliography

[1] Arvestad, L., Berglung, A.C., Lagergren, J., Sennblad, B.: Gene tree reconstruction and orthology analysis based on an integrated model for duplications and sequence evolution. In: Bininda-Emonds, O. (ed.) RECOMB. pp. 238–252 (2004)

[2] Butler, G., Rasmussen, M.D., et al.: Evolution of pathogenicity and sexual reproduction in eight Candida genomes. Nature **459**, 657–662 (Jun 2009). https://doi.org/10.1038/nature08064

[3] Chaudhary, R., Bansal, M.S., Wehe, A., Fernández-Baca, D., Eulenstein, O.: iGTP: a software package for large-scale gene tree parsimony analysis. BMC Bioinformatics pp. 574–574 (2010)

[4] Chaudhary, R., Burleigh, J.G., Fernández-Baca, D.: Inferring species trees from incongruent multi-copy gene trees using the Robinson-Foulds distance. Algorithms for Molecular Biology **8**(1), 1–12 (Dec 2013). https://doi.org/10.1186/1748-7188-8-28

[5] David, L.A., Alm, E.J.: Rapid evolutionary innovation during an archaean genetic expansion. Nature **469**(7328), 93–96 (Dec 2010). https://doi.org/10.1038/nature09649, https://doi.org/10.1038/nature09649

[6] Fiduccia, C., Mattheyses, R.: A linear-time heuristic for improving network partitions. In: 19th Design Automation Conference. pp. 175–181 (June 1982). https://doi.org/10.1109/DAC.1982.1585498

[7] Huerta-Cepas, J., Capella-Gutiérrez, S., Pryszcz, L.P., Marcet-Houben, M., Gabaldón, T.: PhylomeDB v4: zooming into the plurality of evolutionary histories of a genome. Nucleic Acids Research **42**(Database), D897 (Jan 2014). https://doi.org/10.1093/nar/gkt1177

[8] Mahbub, M., Wahab, Z., Reaz, R., Rahman, M.S., Bayzid, M.S.: wQFM: highly accurate genome-scale species tree estimation from weighted quartets. Bioinformatics **37**(21), 3734–3743 (Nov 2021). https://doi.org/10.1093/bioinformatics/btab428

[9] Mahbub, S., Sawmya, S., et al.: Quartet Based Gene Tree Imputation Using Deep Learning Improves Phylogenomic Analyses Despite Missing Data. Journal of Computational Biology **29**(11), 1156–1172 (Nov 2022). https://doi.org/10.1089/cmb.2022.0212

[10] Marcet-Houben, M., Gabaldón, T.: The tree versus the forest: the fungal tree of life and the topological diversity within the yeast phylome. PloS one **4**(2), e4357 (2009)

[11] Markin, A., Eulenstein, O.: Quartet-based inference is statistically consistent under the unified duplication-loss-coalescence model. Bioinformatics **37**(22), 4064–4074 (Nov 2021). https://doi.org/10.1093/bioinformatics/btab414

[12] Mirarab, S., Reaz, R., Bayzid, M.S., Zimmermann, T., Swenson, M.S., Warnow, T.: ASTRAL: genome-scale coalescent-based species tree estimation. Bioinformatics **30**(17), i541–i548 (2014)

[13] Mirarab, S., Warnow, T.: ASTRAL-II: coalescent-based species tree estimation with many hundreds of taxa and thousands of genes. Bioinformatics **31**(12), i44–i52 (06 2015). `https://doi.org/10.1093/bioinformatics/btv234`, `https://doi.org/10.1093/bioinformatics/btv234`

[14] Morel, B., Schade, P., et al.: Speciesrax: a tool for maximum likelihood species tree inference from gene family trees under duplication, transfer, and loss. Molecular biology and evolution **39**(2), msab365 (2022)

[15] Page, R.: Genetree: comparing gene and species phylogenies using reconciled trees. Bioinformatics (Oxford, England) **14**(9), 819–820 (1998)

[16] Rasmussen, M.D., Kellis, M.: Unified modeling of gene duplication, loss, and coalescence using a locus tree. Genome Research **22**(4), 755 (Apr 2012). `https://doi.org/10.1101/gr.123901.111`

[17] Reaz, R., Bayzid, M.S., Rahman, M.S.: Accurate phylogenetic tree reconstruction from quartets: A heuristic approach. PLoS One **9**(8), e104008 (2014)

[18] Salichos, L., Rokas, A.: Inferring ancient divergences requires genes with strong phylogenetic signals. Nature **497**, 327–331 (May 2013). `https://doi.org/10.1038/nature12130`

[19] Snir, S., Rao, S.: Quartets MaxCut: a divide and conquer quartets algorithm. IEEE/ACM Trans. Comput. Biol. Bioinform. **7**(4), 704–718 (2010)

[20] Wehe, A., Bansal, M.S., Burleigh, J.G., Eulenstein, O.: Duptree: A program for large-scale phylogenetic analyses using gene tree parsimony. American Journal of Botany **24**(13), 1540–1541 (2008)

[21] Willson, J., Roddur, M.S., Liu, B., Zaharias, P., Warnow, T.: DISCO: Species Tree Inference using Multicopy Gene Family Tree Decomposition. Systematic Biology **71**(3), 610–629 (08 2021). `https://doi.org/10.1093/sysbio/syab070`, `https://doi.org/10.1093/sysbio/syab070`

[22] Zhang, C., Rabiee, M., Sayyari, E., Mirarab, S.: Astral-iii: Polynomial time species tree reconstruction from partially resolved gene trees. BMC Bioinformatics **19** (05 2018). `https://doi.org/10.1186/s12859-018-2129-y`

[23] Zhang, C., Scornavacca, C., Molloy, E.K., Mirarab, S.: ASTRAL-Pro: Quartet-Based Species-Tree Inference despite Paralogy. Molecular Biology and Evolution **37**(11), 3292–3307 (Nov 2020). `https://doi.org/10.1093/molbev/msaa139`
